# Clinical characteristics and management of immune checkpoint inhibitor‐related pneumonitis: A single‐institution retrospective study

**DOI:** 10.1002/cam4.3600

**Published:** 2020-11-19

**Authors:** Hanping Wang, Yanwei Zhao, Xiaotong Zhang, Xiaoyan Si, Peng Song, Yi Xiao, Xu Yang, Lan Song, Juhong Shi, Haitao Zhao, Li Zhang

**Affiliations:** ^1^ Department of Pulmonary and Critical Care Medicine Peking Union Medical College Hospital Peking Union Medical College and Chinese Academy of Medical Sciences Beijing China; ^2^ Department of Liver Surgery Peking Union Medical College Hospital Peking Union Medical College and Chinese Academy of Medical Sciences Beijing China; ^3^ Department of Radiology Peking Union Medical College Hospital Peking Union Medical College and Chinese Academy of Medical Sciences Beijing China

**Keywords:** checkpoint inhibitor‐related pneumonitis, glucocorticosteroids, immune checkpoint inhibitors, infection, interstitial pneumonitis, re‐challenge

## Abstract

**Introduction:**

The increasing application of immune checkpoint inhibitors (ICIs) will cause more checkpoint inhibitor‐related pneumonitis (CIP), which is a common cause of ICI‐related death. The clinical management of CIP needs further optimization.

**Methods:**

Patients who were managed at Peking Union Medical College Hospital (PUMCH) between February 2017 and December 2019 with a diagnosis of CIP were retrospectively analyzed. Clinical data including clinical manifestations, radiologic data, laboratory and bronchoscopy results, treatments, and outcomes were collected and analyzed. The Mann–Whitney test was used to compare patients with and without co‐infections.

**Results:**

In total, 48 CIP cases in 42 patients were analyzed. The median time from the first dose of ICI to the onset of CIP was 1.9 months (range: 0.1–13.7). Grade 3–4 (G3–4) accounted for 30 cases (71.4%). The most common symptoms were cough (88.1%) and dyspnea (78.6%). The median starting dose of equivalent prednisone (EP) was 55 mg (range: 30–200) for all patients. The median total duration of glucocorticosteroids (GCS) treatment was 42.5 days (range: 15−89). Three patients (7.14%) died because of infection. A higher starting dose and longer duration of GCS (≥30 mg/day; *p* = 0.001) were associated with opportunistic infection. Chest computed tomography (CT) showed diverse and asymmetrical lesions. Twelve patients were re‐challenged, and six patients developed recurrent CIP.

**Conclusions:**

The clinical and imaging manifestations of CIP are various, and differential diagnosis of exclusion is essential. GCS at 1–2 mg/kg is feasible to treat CIP, but the duration of GCS ≥30 mg/day should be used with caution, given the high risk of acquired infections. Re‐challenges of ICI are feasible, but the recurrence of CIP needs to be closely monitored.

## Graphical abstract

This study revealed for the first time the clinical characteristics, diagnosis, and treatment of CIPs in Chinese cancer patients. The recommended starting dose of 1–2 mg/kg GCS is feasible, but the duration of GCS ≥30 mg/day should be controlled, as acquired infections during the GCS treatment rather than refractory CIP could be the main cause of CIP‐related deaths.

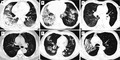

## 1 INTRODUCTION

The development of immune checkpoint inhibitor (ICI) therapy has modified treatment strategies for many malignant tumors, making it a milestone in cancer therapy.^1^, ^2^, ^3^, ^4^, ^5^, ^6^ The principle action of ICIs can be explained as the "brake theory." After releasing the immunological brakes by ICI therapy, unprecedented systemic toxicities, even some refractory and fatal immune‐related adverse effects (irAEs) may occur.^7^, ^8^ ICI can cause checkpoint inhibitor‐related pneumonitis (CIP) in the lungs, which is defined as the occurrence of dyspnea and/or other respiratory symptoms together with new inflammatory lesions on chest computed tomography (CT) following ICI treatment, after the exclusion of pulmonary infection, tumor occurrence, or other reasons.^9^

The incidence of CIP that has been reported in randomized control studies is approximately 4%–6%, most of which are grade 2–3.^10^, ^11^ The actual incidence of CIP may be higher in certain tumor types (non‐small cell lung cancer and renal cell carcinoma), especially in patients treated with combination ICIs, and in non‐trial settings.^12^, ^13^, ^14^ Based on its immunologic mechanisms of action, CIP is considered a special immune mediated interstitial lung disease (ILD).^15^ While the diagnosis and treatment of CIP are primarily recommended according to the experience of ILD, there are significant differences between CIP and classic ILD. Corticosteroid is the basic treatment for CIP, but the most appropriate dose and duration of corticosteroid are unclear according to several available guidelines about irAEs.^16^, ^17^, ^18^ Unfortunately, CIP was the most common cause of treatment‐related death according to the results of a meta‐analysis that included 125 clinical trials involving 20,128 patients.^11^ Some deaths were due to refractory CIP and some because of serious opportunistic infections after immunosuppressive therapy.^15^, ^19^ Therefore, how to optimize management is very important and requires further research.

Because of the low incidence of CIP in patients treated with ICI, a well‐designed large‐sample prospective clinical trial cannot be performed to provide strong evidence; however, retrospective studies from the real world can provide some evidence.^15^, ^19^, ^20^, ^21^

In this retrospective study, we analyzed all CIPs that were diagnosed and treated in the department of Pulmonary and Critical Care Medicine of PUMCH between February 2017 and October 2019. We conducted a detailed review and analysis of their clinical progress in an attempt to explore the optimal treatment for CIP.

## 2 PATIENTS AND METHODS

### 2.1 Patients

This retrospective analysis was conducted on the patients who were managed in PUMCH due to pulmonary inflammatory lesions after ICI therapy between February 2017 and December 2019; patients who were ultimately diagnosed as CIP were included.

The inclusion criteria included: patients who were pathologically diagnosed with locally advanced/advanced cancer; patients who were treated by ICI (PD‐1 inhibitors, PD‐L1 inhibitors, and/or CTLA‐4 inhibitors); and patients who developed new pulmonary inflammation lesions after immunotherapy and were ultimately diagnosed as CIP after evaluation by a multidisciplinary team. The exclusion criteria included: un‐blinded patients in RCT clinical trials for whom it could not be determine whether ICI had been used; patients with other lung diseases with clear etiology; and patients whose data was incomplete or lost to follow‐up. Complete medical records of all included patients were collected.

### 2.2 Methods

The severity of CIP was defined according to the Common Terminology Criteria for Adverse Events, version 4.03.^22^ Detailed clinical data were collected retrospectively, including demographic characteristics, tumor history and prior treatment history, types and antitumor efficacy of ICI, clinical manifestations of CIP, levels of inflammatory factors (C‐reactive protein (CRP), erythrocyte sedimentation rate (ESR), interleukin (IL)‐6, ‐8, and ‐10, and tumor necrosis factor (TNFa)), results of chest imaging and bronchoscopy, and the treatment outcomes of CIP. Data from re‐challenge with ICI and recurrence of CIP were also collected.

Chest CT images obtained at the time of pneumonitis diagnosis were reviewed by a consensus of radiologists with expertise in thoracic and oncologic imaging. CT findings of pneumonitis were evaluated for distributions and specific CT findings including traction bronchiectasis, consolidation, reticular opacities, ground‐glass opacities (GGO), centrilobular nodularity, and interlobular septa thickening. In each case, radiographic patterns of pneumonitis were compared with the classification of interstitial pneumonias according to the ATS/ERS international multidisciplinary approach.^23^

### 2.3 Statistical analysis

Time to the onset of CIP was defined as the time from the first dose of ICI to the first occurrence of CIP‐related symptoms or imaging findings of asymptomatic patients. The data are expressed as *n* (%) for categorical variables and as median (range) for continuous variables. Progression‐free survival (PFS) was estimated using the Kaplan–Meier method with 95% confidence intervals. The Mann–Whitney test was used to compare treatments between patients with and without co‐infections. All reported *p*‐values were two‐sided. For all tests, a statistical difference was considered significant at the 5% level. Statistical analyses were conducted using SPSS Statistics for Windows (Version 19.0; IBM).

## 3 RESULTS

### 3.1 Patient characteristics

There were 60 suspected cases of CIP in 54 patients treated with ICI. After review, a total of 48 CIP cases in 42 patients (six cases were recurrent CIP after ICI re‐challenge) were admitted for analysis (Figure S1). Exclusive reasons included infectious pneumonia, acute exacerbation of COPD, cancerous lymphangitis, pulmonary edema, and radiotherapy‐induced pneumonitis. As a result, CIP accounted for 80.0% (48/60) of pulmonary events after ICI therapy. We analyzed the 42 cases of initial CIPs first.

The general characteristics of these 42 patients are shown in Table 1. The median age was 62 years (range: 29–85). The primary tumors included 17 (40.5%) lung adenocarcinomas, 17 (40.5%) lung squamous cell carcinomas, 3 (7.1%) small cell lung cancers, and 5 (11.9%) hepatic/biliary tract cancer. 33 (78.6%) had a history of smoking, 9 (21.4%) had a prior history of chest radiotherapy (within 6 months), and 7 (16.7%) had a history of lobectomy.

**TABLE 1:** Patient characteristics (N = 42).

Characteristics	Varieties	No.	Frequency
Age, years	Median (range)	62 (29–85)	
	<70	36	85.70%
	≥70	6	14.30%
Gender	Female	5	11.90%
	Male	37	88.10%
Smoking history	Yes	33	78.60%
	No	9	21.40%
Tumor histology	Lung cancer	37	88.10%
	Adenocarcinoma	17	40.50%
	Squamous	17	40.50%
	Small cell	3	7.10%
	Hepatic/Biliary tract cancer	5	11.90%
	Pulmonarymetastasis	2	
Stage of tumor	IIIB–IIIC	11	26.20%
	IV	31	73.80%
History of thoracic radiotherapy	Yes	9	21.40%
	No	33	78.60%
History of pulmonary lobectomy	Yes	7	16.7%
	No	35	83.30%
Line of ICI Treatment	Adjuvant	1	2.40%
	1st line	24	57.10%
	2nd line	11	26.20%
	≥3rd line	4	9.50%
	Maintenance therapy^a^	2	4.80%
Regimen of immune therapy	Monotherapy	17	40.50%
	Combination therapy	25	59.50%
	PD‐1 + Chemotherapy	17	40.50%
	PD‐1 + Antiangiogenesis^a^	6	14.30%
	PD‐1 + ipilimumab	2	4.80%
Best objective response of ICI	Complete/partial response	17	40.50%
	Stable disease	15	35.70%
	Progression of disease	4	9.50%
	Not evaluated	6	14.30%

Abbreviation: ICI, immune checkpoint inhibitor.

^a^ Maintenance therapy after concurrent chemoradiotherapy

^a^ Antiangiogenesis drugs include Anlotinib (2cases), Bevacizumab (1 case), and Lenvatinib (3 cases).

ICI was used as first‐line treatment in 24 (57.1%) patients, as maintenance therapy after concurrent chemoradiotherapy in 2 (4.8%), as second‐line in 11 (26.2%), as third‐line and above in 4 (9.5%), and as adjuvant therapy in 1 (1.7%). Forty (95.2%) patients received PD‐1 inhibitors, and two received PD‐L1 inhibitor. The doses of ICIs are all according to the dosage recommended in their drug instructions.

### 3.2 Clinical features of CIP

**FIGURE 1:**
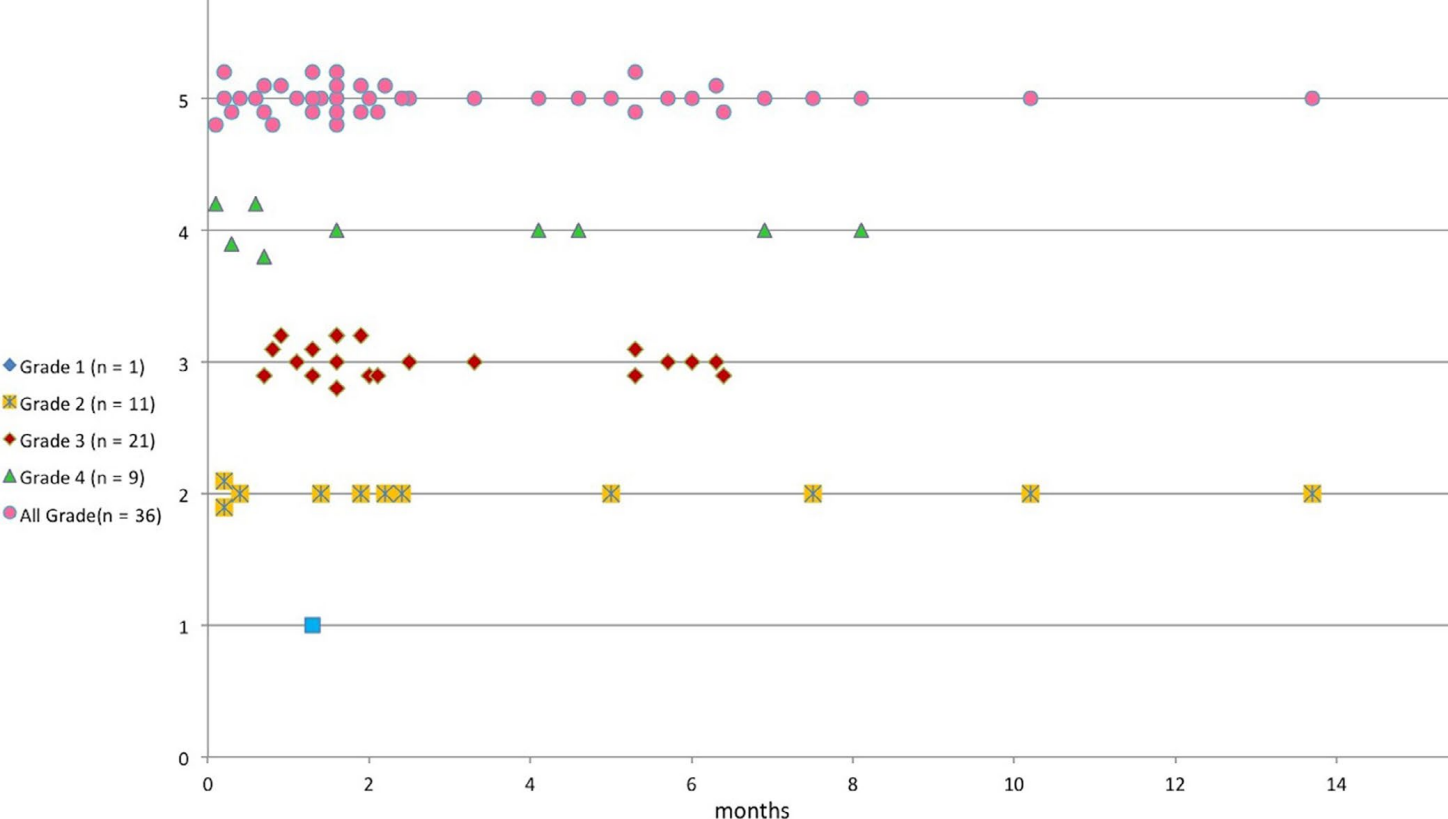
Time from first dose of immune checkpoint inhibitor therapy to date of pneumonitis event stratified by grade.

The median time from the first dose of ICI to the onset of CIP was 1.9 months, with a wide range of 0.1–13.7 months (Figure 1).

According to the CTCAE v4.03 criterion,^13^ 1 (2.3%) patients experienced grade 1, 11 (26.2%) patients experienced grade 2, 21 (50.0%) experienced grade 3, and 9 (21.4%) experienced grade 4 CIP. Severe CIP (grade 3–4) accounted for 71.4% of cases (30 cases).

The most common presenting symptoms of CIP were cough (37, 88.1%) and shortness of breath/dyspnea (33, 78.6%). Seventeen (40.5%) patients had mild to moderate fever. Other symptoms included sputum (6, 14.3%), bloody sputum (2, 4.8%), and chest pain (1, 2.4%); one patient was asymptomatic. Additionally, six (14.3%) patients experienced additional thyroiditis, and one patient showed positive in ANA, anti‐Ro52 antibody, and anti‐SSA antibody after ICI treatment. No other additional irAE was observed.

### 3.3 Inflammatory markers

Routine blood tests showed increased total white blood cells in 37.0% (10/27) of patients and increased neutrophils in 48.1% (13/27) patients, while decreased lymphocyte counts (lowest: 0.3 × 10^9^/L) in 29.6% (8/27) of patients. 92% (23/25) of patients had increased levels of hypersensitive C‐reactive protein (hsCRP), and 91.7% (22/24) showed elevated ESR. The levels of IL‐6, and TNFa were increased in 57.9% (11/19), and 70.6% (12/17) of patients, respectively. The levels of IL‐8 and IL‐10 increased in fewer patients (15.8% (3/19) and 26.3% (5/19), respectively).

### 3.4 Radiological manifestations

The basic lesions of patients with untreated CIP on chest CT included (GGO) in 76.2% (32/42) of patients and consolidation in 54.8% (23/42) of patients. Traction bronchiectasis was found in 33.3% (14/42) of patients, reticular opacities in 28.6% (12/42), centrilobular nodularity in 11.9% (5/42), and interlobular septa thickening in 9.5% (4/42).

Inflammation lesions on chest CT showed an asymmetrical distribution in 64.3% (27/42) of patients, and the remaining 35.7% (15/42) showed a symmetrical distribution. Among the 27 patients with asymmetric lesions, 11 (40.7%) had their inflammatory lesions completely surrounding the tumor lesions, while the remaining 16 showed no relation to tumor lesions.

**FIGURE 2:**
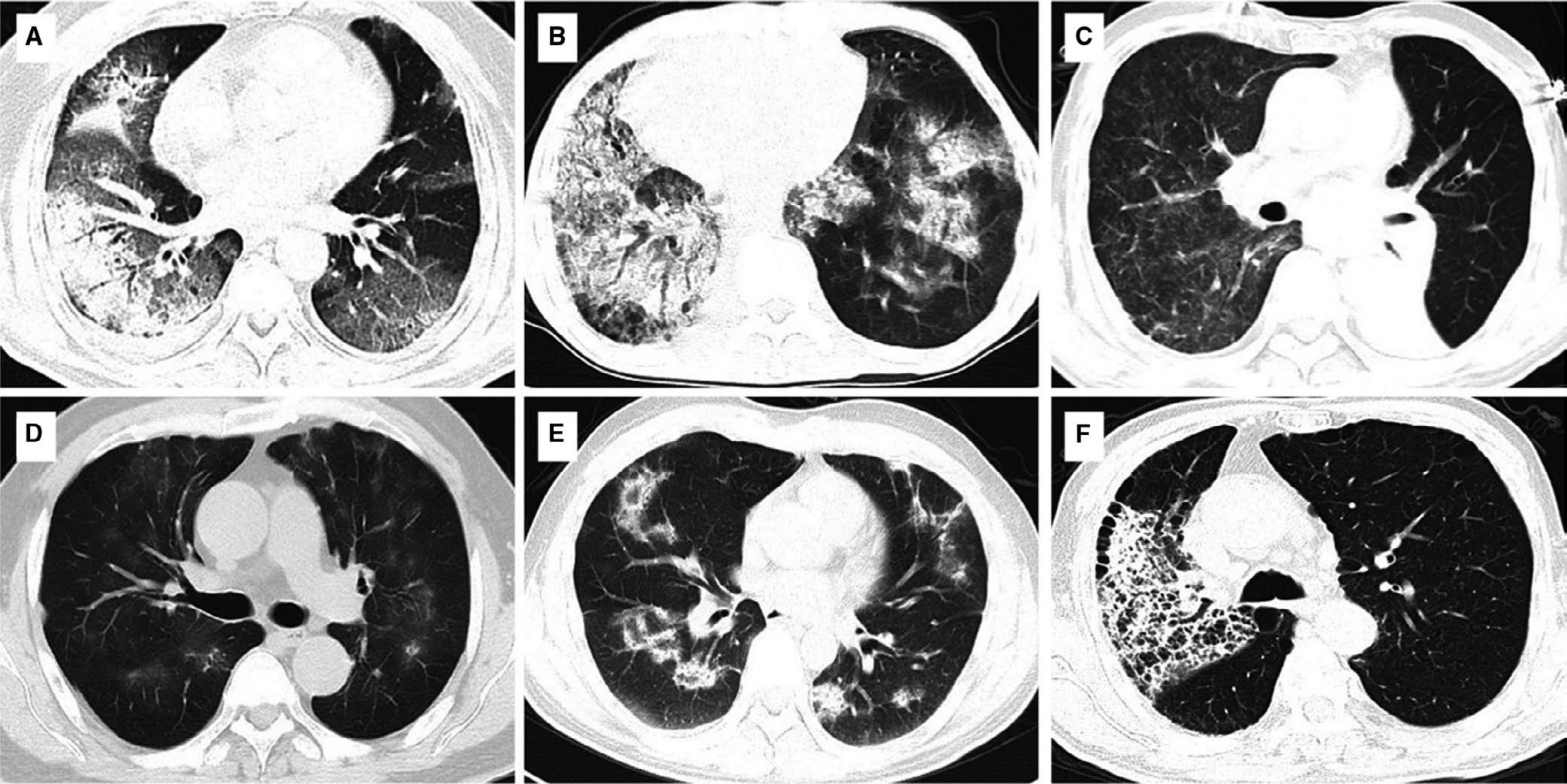
Representative radiological features of immune checkpoint inhibitor‐related pneumonitis. A and B, CT images showing diffuse alveolar damage or acute interstitial pneumonitis, the patients presented with explosive onset and rapid respiratory failure; C, unilateral ground glass opacity; D, multiple ground glass opacities distributed along the bronchovascular bundles, showing hypersensitivity pneumatism like changes; E, multiple consolidation shadows with anti‐halo signs distributed along the bronchovascular bundles, showing cop‐like changes; F, consolidations confined in one lobe of lung, with reticular shadows.

According to the classification of Idiopathic Interstitial Pneumonias imaging patterns, 31.0% (13/42) met the pattern of COP, 14.3% (6/42) met NSIP, 4.8% (2/42) met ARDS/DAD, 4.8% (2/36) met HP, and 42.9% (18/42) were non‐specified. Representative chest CT manifestations are shown in Figure 2.

### 3.5 Bronchoscopy results

Bronchoscopy was performed in 12 patients either before glucocorticosteroids (GCS) treatment (10 patients) or after worsening during GCS treatment (2 patients). A complete description of bronchoscopy results is shown in Table S1.

Bronchoalveolar lavage fluid (BALF) was detected in nine patients before GCS treatment and all cases showed T‐lymphocytic alveolitis. The median percentage of lymphocytes was 52% (range: 11%–95.5%), with a decreased CD4^+^/CD8^+^ (median: 0.6, range: 0.1–1.1), which indicated a predominant increase of CD8^+^ T lymphocytes. In two patients whose symptoms worsened after GCS treatment, bronchoscopy indicated infection, as both of their microbiological evaluations were positive for CMV‐DNA and PCP‐DNA.

### 3.6 Treatment and diagnosis

All patients had stopped ICI treatment definitively. The detailed GCS therapy for each of different grades of CIP is shown in Table 2A. All patients received GCS treatment. Tocilizumab was used in one patient who developed recurrent CIP due to ICI re‐challenge (the level of IL‐6 in the serum of the patient increased significantly) and one patient with Grade 4 CIP (the patient's condition progressed rapidly and had to be intubated soon. He also has fever and elevated hsCRP). No patient received other additional immunosuppressive treatments such as infliximab, cyclophosphamide, or mycophenolatemofetil. The median interval between CIP onset and starting GCS therapy was 7 days (range: 0−31). The median starting dose of EP was 55 mg (range: 30–200). The median time to the first GCS tapering was 7 days (range 3–21). The median total duration of GCS was 42.5 days (range: 15−89).

**TABLE 2:** Management and outcomes of CIP.

CTCAE Grade	Initial oral prednisone	Initial Intravenous MP	Starting dose of equivalent prednisone (mg) Median (range)	Time to taper initiation (days) Median (range)	Duration of GCS dose ≥30 mg (days) Median (range)	Additional immunosuppression	Total duration of GCS Median (range)	Total
A. Management of 35 patients with GCS								
1	1	0	30	7	7	—	42	1
2	8	3	40 (30–50)	7 (7–7)	14 (7–28)	—	42 (28–52)	11
3	6	15	100 (30–200)	7 (3–21)	25 (7–80)	Tocilizumab for 1 patient	49 (15–89)	21
4	0	9	100 (100–200)	7 (7–14)	32 (21–54)	Tocilizumab for 1 patient	46 (32–89)	9
All grade	15	27	55 (30–200)	7 (3–21)	21 (7–80)	—	42.5 (15−89)	42

Patients	CTCAE Grade	Type of initial GCS	Starting dose of equivalent prednisone (mg)	Time to taper initiation (days)	Duration of GCS dose ≥30 mg (days)	Duration from GCS treatment to worsen (days)	Cause of death
B. Management of 3 patients that were died							
1	3	Intravenous MP	150	10	50	22	Infection with CMV and PCP
2	3	Intravenous MP	100	10	80	65	Infection with CMV and PCP
3	4	Intravenous MP	200	7	32	19	Infection

Abbreviations: CIP, Checkpoint inhibitor‐related pneumonitis; CMV, Cytomegalovirus; GCS, Glucocorticosteroids; MP, Methylprednisolone; PCP, *Pneumocystis jirovecii pneumonia*.

**FIGURE 3:**
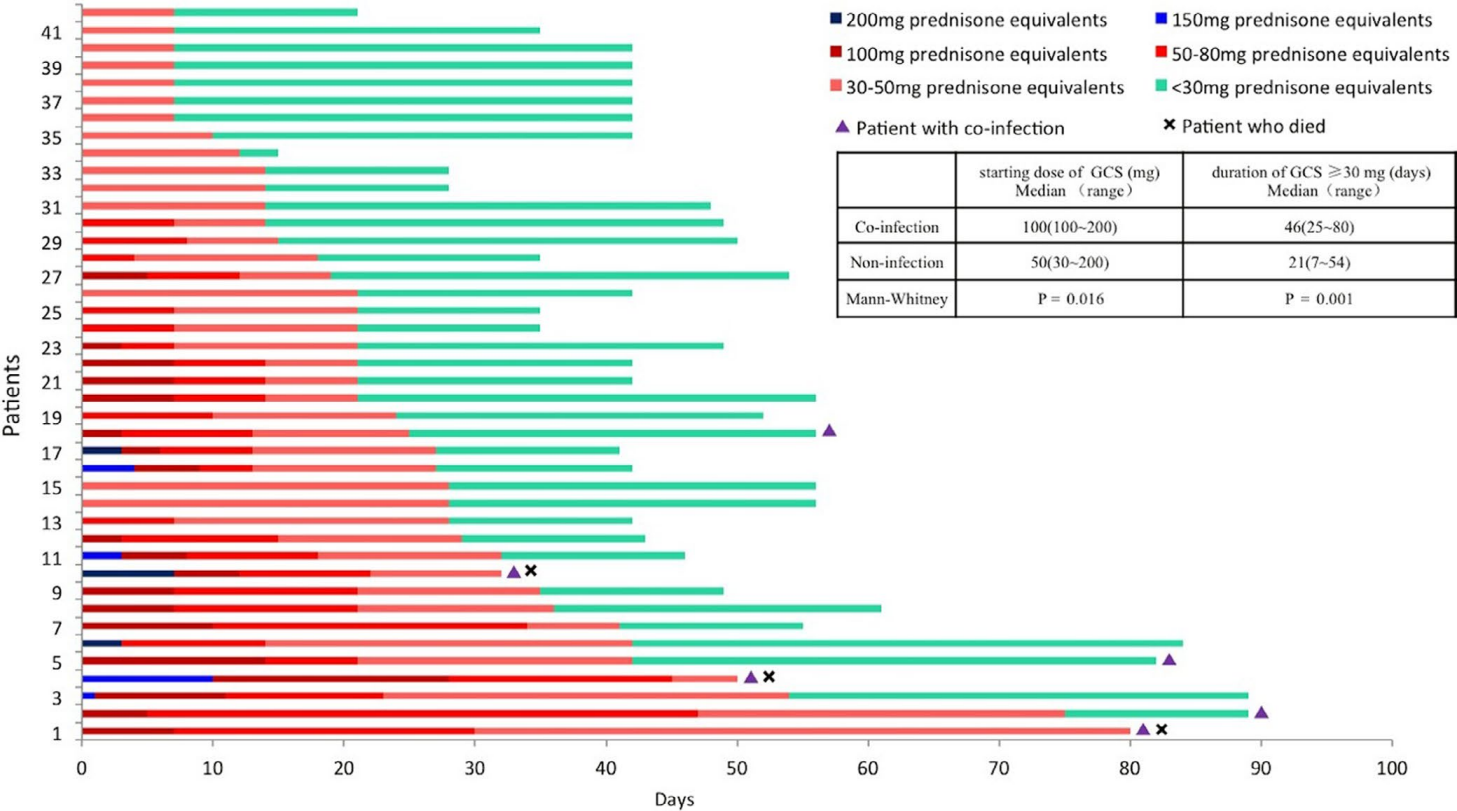
Swimmer’s plot of duration of glucocorticosteroids (GCS) treatment and outcomes of checkpoint inhibitor pneumonitis (CIP). Each bar represents one subject who underwent GCS treatment. A red triangle at the right end indicates a patient who developed an infection after GCS and a black cross indicates a patient who died after GCS therapy. The median starting dose of equivalent prednisone (EP) was 55 mg (range: 30–200). The median total duration of GCS was 42.5 days (range: 15−89). A comparison of patients with and without co‐infection showed that the median duration of GCS ≥30 mg (blue vs. red lines) was significantly longer in patients with co‐infection than in patients without co‐infection (*p* = 0.001)

After GCS therapy, all patients had clinical remission within 2 weeks, and 92.9% (39/42) were totally or partially recovered after GCS withdrawal. However, there were three (7.1%) patients who died of acquired opportunistic infections (Table 2B; Figure 3). No patient died of uncontrolled CIP. There were six (14.3%) acquired infectious pneumonias during GCS treatment (Figure 3). The pathogens included PCP, CMV, bacteria, and unidentified. Compared with patients who did not have co‐infections, the median starting dose of GCS (PE) was significantly higher in those with co‐infection (median: 100 mg, range: 100–200 vs. median: 50 mg, range: 30–200; *p* = 0.016). The duration of GCS ≥30 mg was also longer (median: 46 days, range: 25–80 vs. median 21 days, range: 7–54; *p* = 0.001) (Figure 3). For the three patients who died, their staring doses of GCS were 100, 100 and 200 mg of EP, and their duration of GCS doses ≥30 mg were all >30 days (Table 2B).

### 3.7 ICI re‐challenge and recurrent CIP

Twelve patients were re‐challenged after recovery from initial CIP (seven patients with grade 3 and five with grade 2 initial CIP). Six patients developed recurrent CIP; the comparison of initial and recurrent CIP is shown in Table 3. Four patients accepted the same single ICI for re‐challenging, while for two patients who previously accepted a combined regimen (one combined with chemotherapy and the other combined with CTLA‐4 inhibitor) PD‐1 inhibitor alone was used for re‐challenging. The median interval duration between initial and recurrent CIP was 4.7 months (range: 3.3–20.6). The CIP grade was upgraded in two patients (from G2 to G3) and downgraded in three patients (two downgraded from G4 to G3, and one from G3 to G2). The clinical symptoms appeared different in three patients. CT manifestations varied widely between initial and recurrent CIP in lesion properties (two cases), distribution of sites (two cases), and severity (two cases) (for more detailed imaging findings, see Figure S2). All recurrences were successfully managed after ICI withdrawal and GCS therapy, except for one patient who accepted tocilizumab beyond GCS and finally improved.

**TABLE 3:** Comparison of initial and recurrent CIPs in six patients.

Pts	Immune therapy^@^	Initial CIP					Interval (Ms)	Recurrent CIP				
		Symptom	Grade	CT manifestation	Treatment	Outcome		Symptom	Grade	CT manifestation	Treatment	Outcome
54/M	Pembrolizumab	Dyspnea, cough	3	Diffuse GGO and nodules in both lung	MP 40 mg, tapering, totally 8 weeks	Improved	4.3	Dyspnea, cough	3	Diffuse GGO, nodules, consolidations	MP 80 mg + tocilizumab	Improved
68/M	Pembrolizumab	Dyspnea, cough, fever	4	Diffuse GGO in both lung	MP 80 mg, tapering, totally 8.5 weeks	Resolved	20.6	Dyspnea, cough	3	Diffuse GGO (less severe than the initial CIP)	MP 80 mg, tapering, totally 6 weeks	Resolved
78/M	Pembrolizumab	Chest pain	2	Consolidation in right upper‐middle lobe	Prednisone 30 mg, tapering, totally 8 weeks	Resolved	3.7	Dyspnea, cough, fever	3	Consolidation in left lung	MP 40 mg, tapering, totally 6 weeks	Resolved
64/M	Pembrolizumab	Dyspnea, cough	2	Consolidation and GGO in right Lower lobe	MP 40 mg, tapering, totally 6 weeks	Improved	6.6	Dyspnea, cough	3	Consolidation and GGO in both lungs	MP 40 mg, tapering, totally 6 weeks	Improved
63/M	BGB‐A317+TC^a^	Dyspnea, cough	3	Diffuse centrilobular nodularity/bronchiolitis	Prednisone 40 mg, tapering, totally 6 weeks	Resolved	5.1	Dyspnea, cough	2	Centrilobular nodularity	Prednisone 30 mg, tapering, totally 4 weeks	Resolved
54/M	Pembrolizumab + Ipilimumab^b^	Dyspnea, cough, fever	4	Consolidation, GGO, traction bronchial expansion in both lungs	MP 80 mg, tapering, totally 6 weeks	Resolved	3.3	Dyspnea, cough	3	Diffuse GGO	MP 80 mg, tapering, totally 6 weeks	Resolved

Abbreviations: CIP, Checkpoint inhibitor related pneumonitis; GGO, ground glass opacity; MP, Methylprednisolone.

^a^ The patient accepted BGB‐A317 monotherapy as ICI re‐challenge, TC: paclitaxel plus cisplatin.

^b^ The patient accepted pembrolizumab monotherapy as ICI re‐challenge.

### 3.8 ICI response and survival in patients with CIP

Regardless of the type and specific regimen of ICI, among these 42 patients with CIP, the objective response rate (ORR) of ICI was 47.2% (17/36) and the disease control rate (DCR) was 91.7% (33/36), with six patients un‐evaluable. Twenty‐two patients have not progressed to date. The median PFS was 9.7 months (95% CI: 4.8–14.6) among the 34 patients with NSCLC (16 patients have not progressed yet).

## 4 DISCUSSION

Naidoo et al. first described the clinical, radiologic, and pathologic features of CIP based on data from Memorial Sloan Kettering Cancer Center and The Melanoma Institute of Australia.^15^ Delaunay et al. also reported similar results based on data from several European centers^19^; however, the best treatment for CIP remains unclear and there are no clinical data regarding CIP in Chinese patients.

A CIP diagnosis is not always straightforward, as the symptoms and radiological appearances are not specific for CIP.^9^, ^15^, ^20^ Our data show that even for patients who are highly suspected to have CIP, one fifth of them are finally excluded from CIP. Other reasons, such as infectious pneumonia, acute exacerbation of COPD, cancerous lymphangitis, pulmonary edema, and radiotherapy‐induced pneumonitis could mimic the clinical manifestations of CIP.^9^ It is necessary to make sufficient differential diagnosis to exclude other diseases, and empirical GCS therapy may carry risks of worsen.

CIP can occur at any time after the initiation of ICI treatment. The median onset time of CIP in our cohort was 1.8 months, while the latest onset time was 13.7 months. A whole course monitoring for CIP is needed. In terms of severity, CIP of G3–4 accounted for 71.4% of these cases, and only 1 G1 CIP was included in our cohort, as most patients came to our clinic because of definitive symptoms who need treatment. Oncologist need to do more work on the early diagnosis of CIPs.

Cough and shortness of breath (especially after exercise) were the most common symptom, and fever occurred in 40.5% of patients. Inflammatory factors such as hsCRP and ESR were highly sensitive for inflammatory diseases that were either infectious or non‐infectious. White blood cell and neutrophil counts can also rise in CIP. The lymphocytes in blood usually showed normal or decreased numbers. Other inflammatory factors such as IL‐6 and TNFa can also indicate the inflammatory nature of CIP.

According to the classification criteria of IIP, CIP was first classified into five subtypes according to radiological manifestations: cryptogenic organizing pneumonia (COP)‐like, GGO, interstitial, hypersensitivity, and pneumonitis not otherwise specified.^15^, ^23^ Other retrospective studies have also classified CIP similarly.^19^, ^20^, ^24^ However, a classification‐based solely on imaging features is not accurate and less meaningful to guide treatment or predict outcomes.^25^ Our imaging review indicated that the basic imaging manifestations were dominated by GGO, consolidation, and/or network shadow. An asymmetry distribution is an important characteristic, as more than 60% of patients showed asymmetry lesions on CT. Several patients had the CT manifestations of diffuse alveolar damage or acute interstitial pneumonitis, which often progress rapidly and need more clinical attention and powerful treatment.^26^

GCS is the basic therapy for CIP. However, the exact details for GCS administration are unclear, especially in terms of initial dose, the tapering process, and the overall course.^16^, ^17^, ^18^ The reported mortality rate of CIP is high, suggesting the need to further optimization of treatment.^15^, ^19^, ^27^ Opportunistic infections after GCS therapy may be an important cause of CIP‐related deaths.^11^, ^15^, ^20^ GCS doses of >30 mg have been proven to have significant immunosuppressive effects and are associated with higher risk of infection in rheumatic disease.^28^, ^29^ In our cohort, the starting dose of GCS was <1 mg/kg for CIP of grade 2, and 1–2 mg/kg for CIP of grade 3–4. Almost all patients got benefit from GCS therapy. Three patients died within 3 months, with a mortality rate of 8.3%. The exact cause of death in all three patients was infection. The duration of GCS ≥30 mg/day was significantly longer in patients with opportunistic infections than in those without infection. We suggest that GCS doses should be reduced quickly to <30 mg/day after the initial efficacy of GCS therapy. For patients who need a long duration of GCS ≥30 mg/day, more attention should be paid to the occurrence of infection, and prophylactic anti‐*Pneumocystis jiroveci pneumonia* therapy should be given. For patients who are relieved after GCS therapy but became worse later, infection should be highly suspected first. In addition to finding lymphocytic alveolitis, bronchoscopy can also yield accurate etiological results in patients with suspected infection.^30^

Re‐challenging is feasible in some patients, but there is still a chance of irAE recurrence.^31^ In our cohort, CIP patients of every grade were re‐challenged, and some recurrent CIPs were seen given long enough follow‐up and continued use of ICI. The characteristics of the initial and recurrent CIPs were compared for the first time. The intervals between recurrent CIP were varied, and the clinical manifestations were different, as were the imaging findings including the nature, sites, and severity of the lesions. However, both initial and recurrent CIPs showed good efficacy to GCS therapy. Therefore, for patients who are re‐challenged with ICIs, close and full monitoring for irAEs should be conducted, considering the variety of imaging and clinical manifestations.

The main limitation of this study is the nature of the retrospective single‐center study itself. The sample size is relatively small. However, as all the patients came from a single center, we have very detailed information about the diagnosis and treatment of every case of CIP, and their management was relatively uniform, so the results in term of treatment and outcomes are more reliable than multicenter retrospective studies.

In conclusion, our retrospective study revealed for the first time the clinical characteristics, diagnosis, and treatment of CIPs in Chinese cancer patients. CIP has unique imaging manifestations, and its asymmetry should be noted. The recommended starting dose of 1–2 mg/kg GCS is feasible, but the duration of GCS ≥30 mg/day should be controlled, as acquired infections during the GCS treatment rather than refractory CIP could be the main cause of CIP‐related deaths. Re‐challenges of ICI are feasible, but the recurrence of CIP needs to be closely monitored.

## CONFLICT OF INTEREST

The authors declare that they have no potential conflicts of interest, financial interests, relationships, or affiliations relevant to the subject of their manuscript.

## AUTHORS’ CONTRIBUTIONS

Hanping Wang, Juhong Shi, Haitao Zhao, and Li Zhang contributed to conception and design. Hanping Wang, Yanwei Zhao, Xiaoyan Si, Xiaotong Zhang, Peng Song, Yi Xiao, Xu Yang, Juhong Shi, Haitao Zhao, and Li Zhang contributed to provision of study materials or patients. Hanping Wang, Yanwei Zhao, Xiaoyan Si, Xiaotong Zhang, Peng Song, Yi Xiao, Lan Song, Xu Yang, Juhong Shi, Haitao Zhao, and Li Zhang contributed to collection and assembly of data. Hanping Wang, Yanwei Zhao, Peng Song, Lan Song, Xu Yang, Juhong Shi, Haitao Zhao, and Li Zhang contributed to data analysis and interpretation. All authors wrote the manuscript and approved the final manuscript.

## Supporting information

Fig S1

Fig S2

Table S1

## Data Availability

The manuscript contains the main data, and other data are available at the correspondent author.
