# Supplementary figures and images for: Clinical characteristics and management of immune checkpoint inhibitor‐related pneumonitis: A single‐institution retrospective study
Source: Cancer Med. 2020 Nov 19;10(1):188–98. doi: 10.1002/cam4.3600 (PMC7826478; doi:10.1002/cam4.3600)

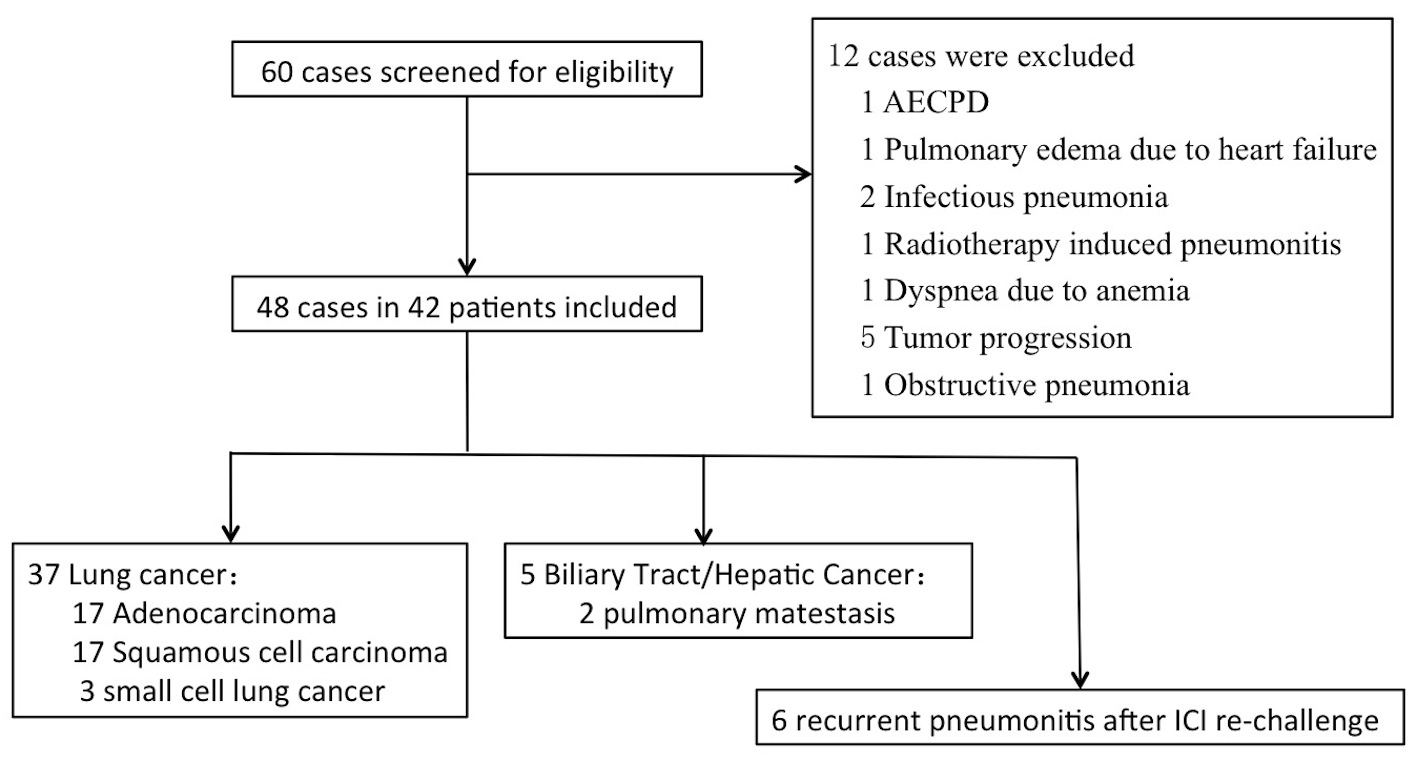

Supplement: Supplementary file 1 — Fig S1 [file CAM4-10-188-s001.jpg]

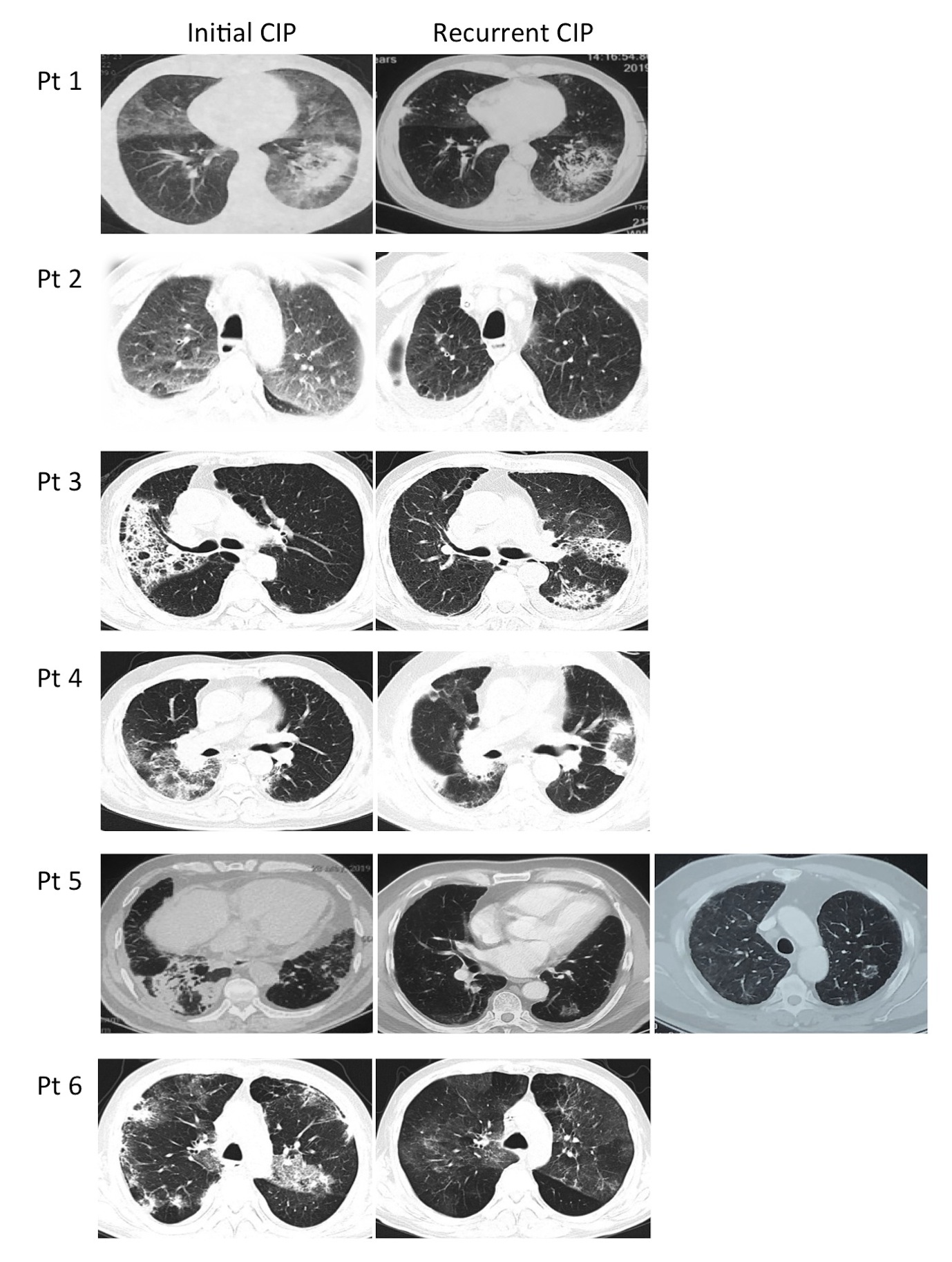

Supplement: Supplementary file 2 — Fig S2 [file CAM4-10-188-s002.jpg]
