# Supplementary material for: Clinical characteristics and management of immune checkpoint inhibitor‐related pneumonitis: A single‐institution retrospective study
Source: Cancer Med. 2020 Nov 19;10(1):188–98. doi: 10.1002/cam4.3600 (PMC7826478; doi:10.1002/cam4.3600)
Supplement: Supplementary file 3 — Table S1 [file CAM4-10-188-s003.docx]

Table 4: Results of BALF and pathogens from bronchoscopy

| No. | Cells/ml | AM％ | N％ | L％ | E％ | CD3+％ | CD4+％ | CD8+％ | CD4／CD8 | Pathogens |
| --- | --- | --- | --- | --- | --- | --- | --- | --- | --- | --- |
| 1 | 981000 | 8.5 | 5.5 | 86 | 0 | 99 | 26.70 | 47.3 | 0.6(🡻) | （－） |
| 2 | 94000 | 80.5 | 0.5 | 19 | 0 | 96.80 | 17.70 | 71.70 | 0.2(🡻) | （－） |
| 3 | 77000 | Unknown | | | | 90.80 | 21.30 | 65.40 | 0.3(🡻) | （－） |
| 4 | 350000 | 15.5 | 10.5 | 67.5 | 6.5 | 97.4 | 43.8 | 47.2 | 0.9 | （－） |
| 5 | 240000 | 51.5 | 13 | 32.5 | 3 | 95.9 | 33 | 57 | 0.6(🡻) | （＋） |
| 6 | 420000 | 48 | 1 | 51 | 0 | 96.90 | 33 | 61.20 | 0.5(🡻) | （－） |
| 7 | 646000 | 29 | 8.5 | 61.5 | 1 | 97.6 | 5.5 | 89.6 | 0.1(🡻) | （－） |
| 8 | 731000 | 2 | 1.5 | 95.5 | 1 | 97.5 | 43.8 | 52.6 | 0.8(🡻) | （－） |
| 9 | 770000 | 42 | 5 | 52 | 1 | 97 | 49.3 | 43.7 | 1.1 | （－） |
| 10 | 181000 | 84 | 1 | 11 | 4 | 96.6 | 17.8 | 75.2 | 0.2(🡻) | （－） |
| 11 | － | － | － | － | － | － | － | － | － | PCP |
| 12 | － | － | － | － | － | － | － | － | － | G-、PCP、CMV |

M :macrophages; L : lymphocytes; N : neutrophil polymorphonuclears; E :eosinophil polymorphonuclears.
